# Hydrogen Peroxide Diffusion through Dental Tissues—In Vitro Study

**DOI:** 10.3390/ma16165552

**Published:** 2023-08-10

**Authors:** Susana Dias, Leonor Casqueiro, Ruben Pereira, João Silveira, António Mata, Duarte Marques

**Affiliations:** 1 Oral Biology and Biochemistry Research Group, Faculdade de Medicina Dentária, Universidade de Lisboa, 1950-044 Lisboa, Portugal; dias.susana@campus.ul.pt (S.D.); leonorcasqueiro@campus.ul.pt (L.C.); ruben-pereira@campus.ul.pt (R.P.); silveira@campus.ul.pt (J.S.); antonio.mata@fmd.ulisboa.pt (A.M.); 2 LIBPhys-FCT UID/FIS/04559/2013, Faculdade de Medicina Dentária, Universidade de Lisboa, 1950-044 Lisboa, Portugal; 3 Postgraduate Programme in Prosthodontics, Faculdade de Medicina Dentária, Universidade de Lisboa, 1950-044 Lisboa, Portugal

**Keywords:** teeth whitening, positive pulpal pressure, hydrogen peroxide diffusion, cytotoxicity

## Abstract

Whitening products commonly utilize hydrogen peroxide (HP) as an active principle, which can penetrate dental tissues with potential side effects due to its low molecular weight. This study aimed to evaluate the HP diffusion of two in-office whitening products, namely 6% VivaStyle Paint On Plus (VS) and Opalescence Boost 40% (OP), in different tooth types. Additionally, the influence of the area of exposure, dental tissue thickness and pulp chamber volume was assessed. Each group consisted of eighteen intact anterior (A), premolar (PM) and molar (M) human teeth, and a positive pulpal pressure model was employed. The samples were analyzed using spectrophotometry, and results were expressed as the mean and 95% confidence interval. Statistical tests and linear regression models were appropriately applied at α = 5%. The total HP (µg) retrieved was as follows: VS-A, 1.333 [1.214, 1.452]; OP-A, 1.538 [1.457, 1.620]; VS-PM, 1.208 [1.123, 1.291]; OP-PM, 3.628 [3.401, 3.855]; VS-M, 2.560 [2.297, 2.823]; and OP-M, 4.197 [3.997, 4.396], with statistically significant differences in diffusion kinetics between whitening products for PM and M. Several HP concentrations attained a minimum cytotoxicity value of 2.22 µg/mL. The regression model shows that OP exposed the pulp chamber to 1.421 µg of HP more than that of VS. Different whitening products can cause cytotoxic HP concentrations in the pulp chamber, with a higher risk observed in molars.

## 1. Introduction

Over the years, the increasing demand for whiter teeth has made teeth whitening the most popular elective dental procedure [1,2,3]. It is a minimally invasive and easily accessible treatment that relies mainly on active ingredients such as hydrogen peroxide (HP) and carbamide peroxide (CP) [1,2,3,4,5]. Due to its increased structural stability and sustained action when associated with carbopol and glycerin, CP is usually used in at-home bleaching techniques at concentrations ranging between 10 and 16% [1,2,6,7]. When in contact with water, a solution of 10% CP releases oxygen, dividing itself into HP (3.35%) and urea (6.66%) [1,2]. In contrast, HP, which is less structurally stable and hydrolyzes more rapidly, dissociates into water and free oxygen radicals. As a result, it serves as the primary active principle in in-office bleaching techniques, with concentrations ranging from 5% to 40%, aiming for faster results [1,2,6,7].

According to the European Union’s Directive [8], teeth whitening can be performed using over-the-counter products containing 0.1% HP or less and through in-office and ambulatory techniques with products containing 0.1–6% HP or equivalent, qualified as cosmetics, or more than 6% HP, marketed as medical devices, both requiring dispensation by dental professionals.

Hydrogen particles and free oxygen radicals can degrade chromogens on the tooth surface. However, due to their low molecular weight, these molecules can diffuse through the dental tissues to the pulp chamber, potentially causing cytotoxic effects [6,9,10]. Consequently, teeth whitening can lead to histologic and clinical side effects, including dental sensitivity [9,11,12,13], DNA damage in odontoblasts [14,15,16], changes in enamel and dentin [9,11,17], decreased adhesive properties of restorative dental materials [9,11,18,19,20], soft tissue ulceration [21], and odontoblast alterations [22,23,24], which decrease cellular metabolism and viability [21,22,23,25,26,27].

Two main factors decrease the molecular diffusion through the dental tissues into the pulp: positive pulpal pressure and the osmotic pressure of the whitening product [21]. Furthermore, even in products with similar HP concentrations, the diffusion and the biological effects may vary depending on the brand and composition of the product [25]. Although research has investigated HP diffusion through intact dental tissues [28,29,30,31,32,33,34], only two pilot studies have aimed to provide in vitro models that simulate in vivo conditions, including positive pulpal pressure [35,36]. Due to blood flow, pulpal tissues apply positive pressure on the remaining dental structure [37]. In a study by Ciucchi in 1995 [37], positive pulpal pressure was simulated on intact human teeth, and fluid movement through the dentin was measured under exogenous pressure. Ciucchi concluded that positive pulpal pressure is equivalent to the pressure generated by a 14 cm column of water [37]. Therefore, it is important to evaluate the diffusion of the HP present in different whitening products at different concentrations using a positive pulpal pressure model.

The present study aims to evaluate the diffusion of HP from two in-office whitening products (6% and 40% HP) into the pulp chambers of different tooth types. The null hypothesis being tested is as follows: There are no differences in HP diffusion kinetics between two whitening products with different HP concentrations. The secondary objectives are to evaluate the diffusion kinetics in different types of teeth and to assess the influence of exposure area, pulp chamber volume and dental tissues’ thickness on the HP diffusion kinetics of two whitening products with different HP concentrations.

## 2. Materials and Methods

In this study, two in-office whitening products were used: a paint-on varnish with 6% HP (VivaStyle Paint On Plus [VS], Ivoclar Vivadent^®^, Schaan, Liechtenstein) and a gel with 40% HP (Opalescence Boost [OP], Ultradent^®^, South Jordan, UT, USA). The manufacturers’ claimed HP concentrations were verified in both products through titration, using the previously described Cerium IV Sulfate (CS, CeSO_4_·4H_2_O, # 31606, Sigma-Aldrich, Darmstadt, Germany) technique [35,38]. To perform the titration, 500 mg of the product was dissolved in 225 mL of distilled water, and 25 mL of a 1:5 diluted sulfuric acid solution was added. The resulting solution was agitated, and 5 mL of an indicator ferrocene solution (Fluka, Buchs, Switzerland) was added, causing the solution to turn orange. Each product sample was titrated until the equivalence point was reached, indicated by a light blue color of the solution. The volume of CS (1 mol/dm^3^) required to reach the equivalence point was used to calculate the percentage of HP present in each sample [39]. This technique was performed three times for each whitening product to acquire multiple measurements. The relationship between the volume of CS used in the titration of the sample and the weight of the sample was incorporated into a formula to determine the percentage of HP (% by weight) recovered in each sample (Equation (1)):

%H_2_O_2_ (w/w) = (CS Volume (mL) ∗ 0.17)/sample weight (g) (1)

### 2.1. Tooth Sample and Positive Pressure Model Preparation

**Scheme 1:**
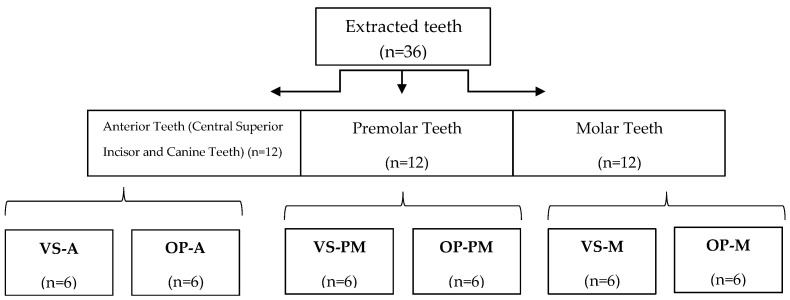
Sample distribution. VivaStyle Paint On Plus varnish system with 6% HP (VS) 6× 10-min applications at room temperature. Opalescence Boost gel system with 40% HP (OP) 3× 20-min applications at room temperature.

The selected teeth were from the duly approved faculty teeth repository, in which the teeth are collected at the University Clinic, and had been stored in a chloramine-based solution in a cold and dry environment for a period of up to 6 months. From patients between 16 and 62 years of age, anterior teeth, which were upper central incisor and canine teeth, were extracted for periodontal reasons; premolars were extracted for orthodontic reasons; and molar teeth, which were third molars, were extracted for therapeutic reasons. A total of thirty-six teeth without caries, restorations or any development anomalies were used and distributed according to the description provided in Scheme 1. Six teeth of each type—anterior, premolar and molar—were randomly allocated to each whitening product using established software (GraphPad QuickCals. © 2021 GraphPad Software. http://www.graphpad.com/quickcalcs/randomize1.cfm (accessed on 24 September 2021)).

Using a precision saw (IsoMet 1000 Precision Saw—Buehler^®^, Lake Bluff, IL, USA), the sampled teeth were horizontally sectioned approximately 2–3 mm below the cementoenamel junction, the canal was enlarged using a round bur, and the radicular portion was discarded. The pulpal tissue was then carefully removed with a small spoon excavator.

**Figure 1:**
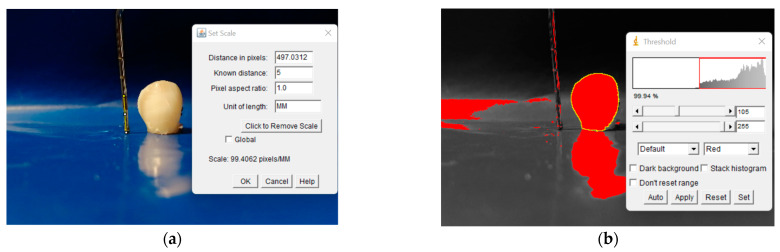
(**a**) A periodontal probe was used to provide a known distance and to set the scale. (**b**) The photograph was transformed into an 8-bit file. The desired area was automatically selected before measuring.

The volume of the pulp chamber was determined for each sample by subtracting the tooth’s weight with an empty pulp chamber from the tooth’s weight with its pulp chamber filled with distilled water. Additionally, the thickness of the buccal surface was determined using a thickness gauge to measure the cervical, buccal and incisal/occlusal dimensions of each sample. Furthermore, the exposed area of the buccal surface was determined according to three measures (Figure 1) using a software program to calculate irregular areas based on a previously set scale (ImageJ^®^, v 1.53m 2021, Bethesda, MD, USA).

**Figure 2:**
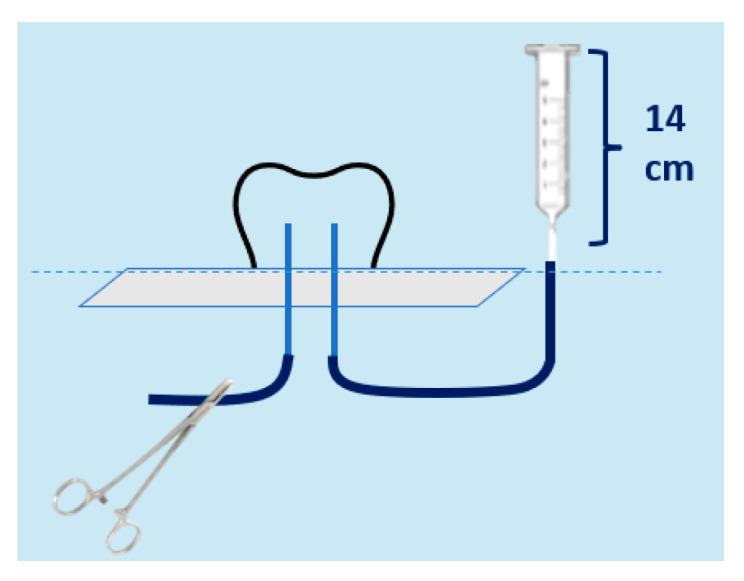
Representation of a positive pulpal pressure model.

The tooth samples were affixed to polycarbonate plates using cyanoacrylate adhesive (SuperCola 3^®^, Loctite, Henkel Adhesives, Aachen, Germany). These plates were drilled twice to establish communication with the internal part of the pulp chamber by inserting 27G needles (Luer Lock ^®^, B. Braun Melsungen, Melsungen, Germany). Every surface of the sample was coated using nail polish (Kiko Milano^®^, Milan, Italy), except for the buccal surface, which was left exposed for the application of the bleaching product. An entry tube was connected to a 14 cm-tall column of sodium acetate (2 mol/dm^3^) buffer solution with a pH of 4.5, enabling the establishment of a positive pulpal pressure model (Figure 2) [37,40,41]. The exit tube was sealed with a needle holder while applying the whitening product and removed when buffer samples were required.

### 2.2. Buffer Samples Collection

Following the manufacturer’s instructions, the product was applied at 0, 10, 20, 30, 40, 50 and 60 min in groups VS-A, VS-PM and VS-M. In groups OP-A, OP-PM and OP-M, the product was applied at 0, 20 and 40 min. Samples (0.125 mL) were collected every 10 min during the product application and 30 min after the last application. Before applying the product, a buffer sample was collected through the system to establish the baseline value for each tooth sample. The whitening products were applied to the buccal surface of the tooth samples according to the manufacturer’s instructions. The applied amount was determined by weighing the product container before and after each application. Before applying a new layer of product, the tooth samples were cleaned with distilled water and a cotton pellet.

The diffused HP concentration in the pulpal buffer was measured using a spectrophotometer (M501 single beam, CAMSPEC, Leeds, West Yorkshire, UK) at a wavelength of 596 nm at room temperature. Following the technique described by Mottola 1970 [42], the absorbances of 10 standard samples with different concentrations of HP (ranging from 0 to 0.65 µg/mL) were measured to calibrate the spectrophotometer. For each collection, a falcon tube was filled with a 0.125 mL extract from each tooth sample and mixed with 2 mL of sodium acetate buffer solution (2 mol/dm^3^, pH 4.5). Then, 0.5 mL of leucocrystal violet solution (0.5 mg/mL; Sigma-Aldrich Corporation, St. Louis, MO, USA), 0.25 mL of horseradish peroxidase solution (1 mg/mL; Sigma-Aldrich Corporation, St. Louis, MO, USA) and 2.125 mL of distilled water were added. The falcon tubes were vortexed, and the mixture was transferred to microcuvettes and measured in the spectrophotometer.

The results were compared with the minimum cytotoxic concentrations (MCC) for odontoblasts established by De Lima, 2009 [27] (2.22 µg/mL) and Almeida 2013 [26] (4.70 µg/mL) to infer the toxic potential of HP. The 50% inhibitory dose (ID_50_) of HP for succinyl dehydrogenase activity in cultured cells for odontoblasts determined by Hanks, 1993 [21] was also considered −0.58 mmol/L, which is equivalent to 19.73 µg/mL.

### 2.3. Statistical Analysis

The statistical analysis was performed with IBM SPSS^®^ (version 25) (IBM Statistics, Inc. Chicago, IL, EUA). It included the following analyses: a sample *t*-test to compare titration values to the manufacturer’s HP percentage; the Kolmogorov–Smirnov test to assess the normality of the distribution of each variable; a two-sample test with unequal variance to evaluate whether the mean HP diffusion for each time slot and tooth type was independent of the product used; Pearson correlations to determine the influence of area, volume and thickness on diffused HP weight, classified as negligible (0–0.10), weak (0.10–0.39), moderate (0.40–0.69), strong (0.70–0.89) and very strong (0.9V1) correlations [43]; the Kruskal–Wallis test to determine the influence of tooth type on diffused HP weight; and linear regression models. Results were presented as mean values with a confidence interval (CI) of 95% and a significance level established at α = 0.05.

## 3. Results

### 3.1. Titration

Three titration measurements were performed for each whitening product. The VS varnish system had an average HP percentage of 6.153% [5.927, 6.378], and the OP gel system had an average HP percentage of 41.043% [37.587, 44.514], without statistical differences from the reference values reported by the manufacturers.

### 3.2. HP Diffusion Kinetics

**Figure 3:**
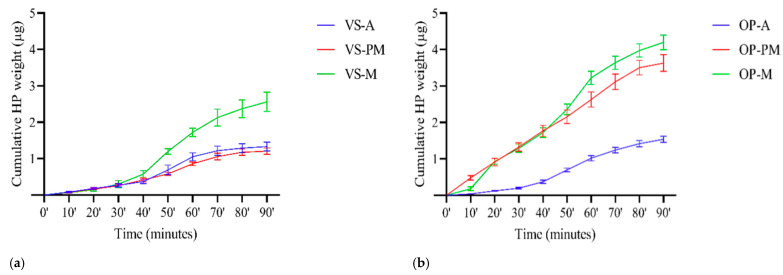
(**a**) Mean cumulative recovered hydrogen peroxide (HP) weight (µg) with 95% CI for the VivaStyle Paint On Plus (VS) application groups. (**b**) Mean cumulative recovered HP weight (µg) with 95% confidence intervals for Opalescence Boost (OP) application groups.

The cumulative total HP after 90 min was 1.333 µg [1.214, 1.452] for the VS-A group and 1.538 µg [1.457, 1.620] for the OP-A group. The VS-PM and the OP-PM exhibited a mean total retrieved HP of 1.208 µg [1.123, 1.291] and 3.628 µg [3.401, 3.855], respectively, with statistically significant differences between the groups. The VS-M and OP-M groups had values of 2.560 µg [2.297, 2.823] and 4.197 µg [3.997, 4.396], respectively, with statistically significant differences between the groups (Figure 3a,b).

Regarding comparisons within the same whitening product, there were statistically significant differences in the diffusion kinetics of VS-A and VS-PM compared to those of VS-M. Conversely, there were statistically significant differences in the diffusion kinetics of OP-PM and OP-M compared to those of OP-A.

The maximum diffusion of HP occurred between 50 and 70 min for all groups, regardless of the product and tooth type. The highest value was observed in the OP-M group at 60 min (0.865 µg) (Table 1).

**Figure 4:**
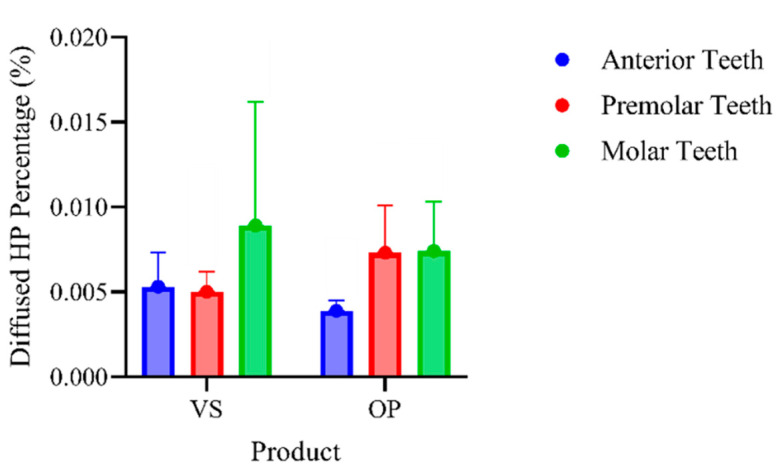
Mean percentage of recovered hydrogen peroxide (HP) (%) and upper limit of the 95% confidence interval.

Ninety minutes after the start of the whitening product application protocol, the percentage of retrieved HP ranged from 4.999 × 10^−5^% to 8.877 × 10^−3^% in the VS groups. Moreover, the OP groups registered an HP diffusion of 3.866 × 10^−3^% to 7.377 × 10^−3^% (Figure 4).

Pearson correlations were determined between the diffused HP total weight and three variables: the area of exposure, the pulp chamber volume and the thickness of the buccal surface. The variables showed positive correlations, with the strongest correlation observed between the diffused HP total weight and pulp chamber volume. The correlations for the area of exposure, the pulp chamber volume and the thickness of the buccal surface were 0.077, 0.536 and 0.312, respectively. All correlations were positive, but none were statistically significant (*p* > 0.05).

**Figure 5:**
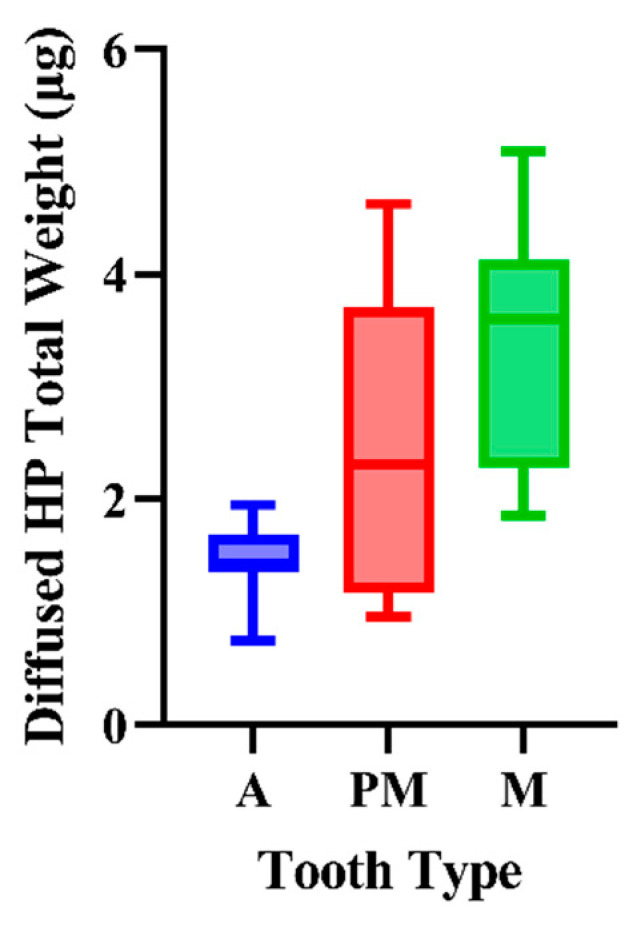
Box plot representation of the diffused hydrogen peroxide (HP) total weight (µg) by tooth type, blue for anterior teeth, red for premolar teeth, and green for molar teeth.

The Kruskal–Wallis test demonstrated that the type of tooth had a significant influence on the median HP obtained from the pulpal chamber (Figure 5).

Multiple linear regression models were developed to evaluate the correlation between the recovered HP weight (dependent variable) and the whitening product applied, the tooth type and one of the following variables: the area of exposure, the pulp chamber volume or the thickness of the buccal surface. The model that assessed tooth type in relation to the whitening product showed statistically significant results for all variables. Hence, the following Equation (2) was estimated, where PMx, My and OPz are dummy variables representing whether the tooth is premolar or molar, and whether or not the whitening product is OP, respectively, for the calculation of diffused HP total weight (µg) 90 min after the start of the application protocol with OP or VS in anterior, premolar and molar teeth:

Diffused HP total weight (µg) = 0.725 + 0.982(PMx) + 1.943(My) + 1.4210(OPz)(2)

This model explains 72% of the variability verified in the diffused HP weight. This equation suggests that, after the application of the whitening product, there is an average diffusion of an additional 0.482 µg and 1.943 µg of HP to the pulpal chamber in premolar and molar teeth, respectively, compared to anterior teeth. The application protocol of the OP whitening product is also expected to lead to the diffusion of an additional 1.421 µg of HP compared to the VS whitening product’s application protocol, independently of the tooth type.

### 3.3. HP Concentrations in the Pulp Chamber

**Figure 6:**
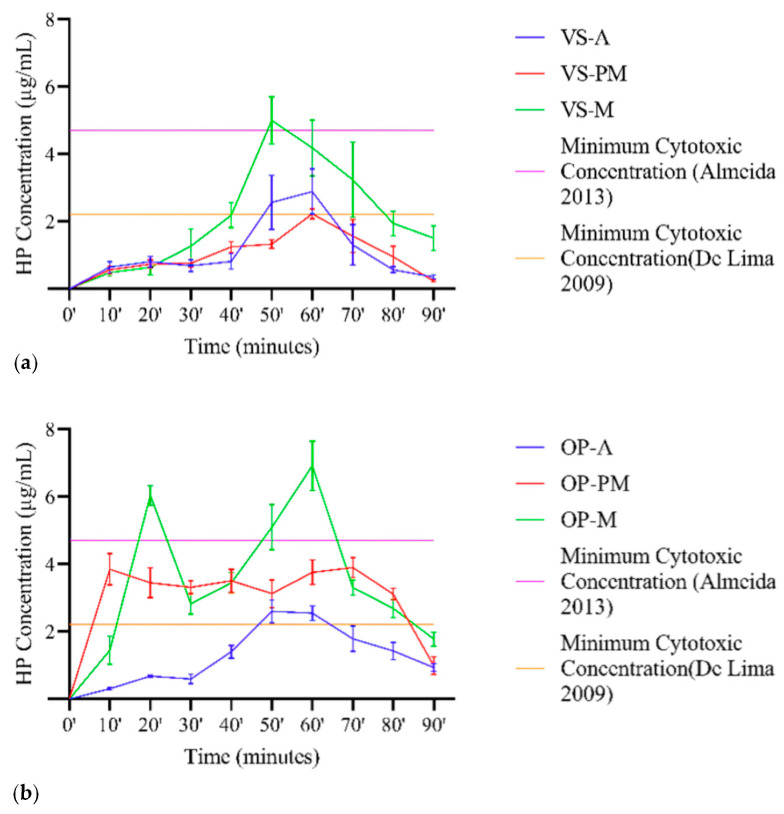
(**a**) Mean hydrogen peroxide (HP) concentration (µg/mL) per tooth type for VivaStyle Paint On Plus (VS) groups and 95% confidence interval (CI). (**b**) Mean hydrogen peroxide (HP) concentration (µg/mL) per tooth type for Opalescence Boost (OP) groups and 95% CI.

The HP concentration (µg/mL) in the pulp chamber for each time slot was determined based on the diffused HP weight (µg) and sample volume (mL) (Figure 6).

## 4. Discussion

This study assessed the in vitro diffusion kinetics of hydrogen peroxide (HP) in two in-office whitening products and investigated the influence of tooth type, area of exposure, pulp chamber volume and dental tissue thickness. The findings of this study demonstrate significant differences in HP diffusion kinetics between the two tested whitening products, thereby rejecting the null hypothesis.

HP diffusion kinetics toward the pulp chamber appeared to be dependent on the bleaching product used and the type of tooth, with significant differences in the cumulative amount of retrieved HP between premolar and molar teeth. Although previous studies have analyzed HP diffusion in intact human teeth, only two pilot studies have considered positive pulpal pressure to mimic in vivo conditions [35,36]. Nonetheless, despite being the first study to evaluate differences between tooth types, the existing literature with a similar methodology for assessing HP diffusion kinetics toward the pulp chamber aligns with our results. Notably, Gokay, 2005 [31] and Bharti, 2013 [33] used central maxillary incisors and employed 30 min application protocols (with formulations containing 5.3% to 8.7% HP or equivalent), resulting in HP amounts ranging from 0.175 µg to 0.398 µg. In contrast, Kwon, 2012 [28] and Cardoso, 2015 [35] utilized canine teeth and applied a 40% HP product for 60 min (three 20 min applications), registering diffused HP weights of 0.8 to 0.93 µg. Finally, Vieira, 2018 [34] employed third molars and applied a product with 38% HP, resulting in a diffusion of 1.206 µg after 45 min. In our study, the HP diffusion observed for the same tooth types and application time was 0.268 µg, 1.012 µg and 1.723 µg for anterior, premolar and molar teeth, respectively, suggesting an increased HP diffusion in posterior teeth. The diffusion area in dentin is directly influenced by the density and diameter of dentinal tubules, which increases with pulp chamber proximity. Additionally, as the thickness of dentin decreases, its permeability increases. These factors can regulate the movement of substances within the teeth during teeth whitening [25,26,44,45].

Although VS and OP have different initial HP concentrations, the cumulative diffused HP does not reflect this ratio. A varnish system (such as VS) is composed of a cellulose matrix and an ethanolic base, and for the matrix to solidify, the ethanolic base must evaporate, which allows for a higher superficial concentration of HP, a steeper concentration gradient and an increased diffusion rate compared to what is expected [46,47,48].

The differing time patterns observed, with statistically significant differences per tooth type, may be attributed to variations in the area of exposure, the thickness of the buccal surface and, most likely, the pulp chamber volume, which showed a strong correlation with the diffused HP weight. However, a larger sample size is required to accurately infer the influence of these variables on the diffused HP weight.

The proposed multiple regression model confirmed that premolar and molar teeth exhibited greater diffusion compared to anterior teeth. These differences could be due to the permeability of dental tissues, which is affected by factors such as tooth eruption time and periodontal condition and directly influences the movement of HP into the pulp chamber. Although the absolute pulp chamber volume provides information about the physical space within the tooth, and the positive pulpal pressure adds one factor present in in vivo conditions that justifies the apparent discrepancy between in vitro results and clinical symptoms, it does not account for the complexities of tissue permeability and the physiological variability [25,35]. The anterior teeth used in this study were extracted for periodontal reasons, the premolars were extracted for orthodontic reasons, and the third molars were extracted for therapeutic reasons, which could have also influenced the results. Additionally, diffusion through young dentin is significantly higher than that through old dentin, and these biological factors are fundamental determinants of how well hydrogen peroxide can diffuse through the dental structures and reach the pulp chamber [49]. Moreover, the OP protocol resulted in an additional 1.421 µg of HP diffusion compared to VS, regardless of tooth type, after 90 min, which can be attributed to the difference in HP content.

With respect to cytotoxicity levels, all groups exceeded the MCC defined by De Lima, 2009 [27] at some point in time, and HP diffusion was detected even after the completion of the application protocol, indicating cumulative cytotoxic potential in the case of consecutive applications. However, none of the concentrations obtained surpassed the ID_50_ value stated by Hanks, 1993 [21]. The correlation assessments indicated that the pulp chamber volume had the strongest correlation with cytotoxicity. Therefore, caution should be exercised when treating younger patients, as they have thinner dentin and larger pulp chambers, which increases the risk of cytotoxicity and pulp cell alterations [49].

Nevertheless, it is essential to consider that in vitro studies may have limited value in simulating clinical conditions and that, in vivo, the pulp has mechanisms that protect the tissue from the free radicals generated from HP, which could potentially reduce cytotoxicity exposure [45,50].

Further in vitro studies should incorporate different whitening products and HP concentrations while focusing on specific sample groups (e.g., only central maxillary incisors or mandibular premolars, and taking into account the age of the patient and periodontal condition of the teeth). Moreover, future clinical studies should investigate which tooth type is more susceptible to post-whitening symptomatology.

## 5. Conclusions

The two systems presented differences in the HP diffusion kinetics for the pulp chamber for all types of teeth. Diffused HP values varied with the whitening product. Namely, the proposed model for calculating the diffused HP total weight suggests that OP leads to diffusion into the pulpal chamber of an additional 1.421 µg of HP compared to the VS whitening product’s application protocol, independently of the type of tooth. Moreover, the area of exposure, the buccal surface’s thickness and the pulp chamber volume of the samples have a non-significant positive correlation with the final diffused HP.

The findings of this study demonstrate that odontoblasts may be exposed to cytotoxic concentrations of HP and free oxygen radicals during teeth whitening, with molars being the tooth type at the highest risk.

## Figures and Tables

**Table 1:** Mean recovered hydrogen peroxide weight (µg) and 95% confidence interval per time slot (minutes).

Time (Minutes)	VS-A (n = 6)	OP-A (n = 6)	VS-PM (n = 6)	OP-PM (n = 6)	VS-M (n = 6)	OP-M (n = 6)	VS Overall (n = 18)	OP Overall (n = 18)
10′	0.081 [0.060, 0.102]	0.032 [0.028, 0.036]	0.071 * [0.058, 0.084]	0.482 * [0.423, 0.540]	0.062 [0.049, 0.075]	0.182 [0.130, 0.234]	0.071 * [0.062, 0.080]	0.234 * [0.189, 0.279]
20′	0.101 [0.081, 0.121]	0.085 [0.080, 0.090]	0.093 * [0.078, 0.108]	0.432 * [0.376, 0.487]	0.080 * [0.053, 0.107]	0.755 * [0.717, 0.793]	0.091 * [0.079, 0.103]	0.424 * [0.365, 0.483]
30′	0.086 [0.065, 0.107]	0.075 [0.058, 0.092]	0.095 * [0.080, 0.110]	0.415 * [0.391, 0.439]	0.158 [0.093, 0.223]	0.354 [0.315, 0.393]	0.113 * [0.090, 0.136]	0.281 * [0.248, 0.315]
40′	0.103 [0.075, 0.131]	0.176 [0.152, 0.200]	0.157 * [0.137, 0.177]	0.438 * [0.395, 0.481]	0.274 [0.227, 0.321]	0.432 [0.394, 0.470]	0.178 * [0.154, 0.201]	0.349 * [0.317, 0.380]
50′	0.321 [0.221, 0.421]	0.325 [0.283, 0.367]	0.167 * [0.151, 0.183]	0.391 * [0.340, 0.442]	0.625 [0.538, 0.712]	0.638 [0.554, 0.722]	0.371 [0.314, 0.428]	0.451 [0.408, 0.495]
60′	0.362 [0.279, 0.445]	0.319 [0.292, 0.346]	0.279 * [0.260, 0.298]	0.470 * [0.425, 0.515]	0.523 [0.420, 0.626]	0.865 [0.774, 0.956]	0.388 [0.340, 0.435]	0.551 [0.494, 0.609]
70′	0.164 [0.090, 0.238]	0.224 [0.177, 0.271]	0.197 * [0.137, 0.257]	0.488 * [0.450, 0.525]	0.405 [0.265, 0.545]	0.413 [0.385, 0.441]	0.255 [0.197, 0.314]	0.375 [0.344, 0.406]
80′	0.072 * [0.061, 0.083]	0.178 * [0.146, 0.210]	0.120 * [0.081, 0.159]	0.389 * [0.367, 0.411]	0.243 [0.197, 0.289]	0.336 [0.302, 0.370]	0.145 * [0.121, 0.169]	0.301 * [0.277, 0.326]
90′	0.045 * [0.037, 0.053]	0.118 * [0.103, 0.132]	0.030 * [0.026, 0.034]	0.124 * [0.091, 0.157]	0.189 [0.144, 0.234]	0.223 [0.196, 0.250]	0.088 * [0.068, 0.109]	0.155 * [0.138, 0.172]

* Null hypothesis is rejected.

## Data Availability

Original data are available on request from the corresponding author.
